# Supplementary material for: Photophysical Properties of Multilayer Graphene–Quantum Dots Hybrid Structures
Source: Nanomaterials (Basel). 2020 Apr 9;10(4):714. doi: 10.3390/nano10040714 (PMC7221764; doi:10.3390/nano10040714)
Supplement: Supplementary file 1 [file nanomaterials-10-00714-s001.pdf]

Photophysical properties of "Multilayer Graphene Nanobelts - QDs" hybrid structures
Supplementary Information

S1. QD optical properties

According to Ref. [1], the use of OA molecules as QD stabilizer allows forming large surface monolayers of QDs by L-B method. Therefore, initial QD stabilizer molecules, i.e. a mixture of TOPO with OA molecules, were replaced by OA (see Section 1.2 for details). Figure S1 presents (a) FTIR and (b) absorption and PL spectra of studied CdSe/ZnS QDs before and after replacement of the initial QD stabilizing molecules by OA.

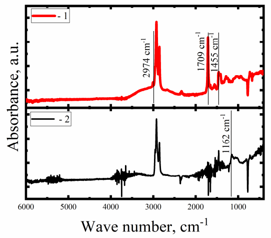

(**a**)

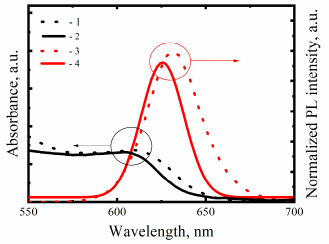

(**b**)

Figure S1. (a) FTIR spectra of studied CdSe/ZnS QDs before (1) and after (2) replacement of the initial QD stabilizing molecules by OA. (b) absorption (1,2) and PL (3,4) spectra of CdSe/ZnS QDs before (1 and 3) and after (2 and 4) replacement of the initial QD stabilizing molecules by OA.

Analysis of the FTIR spectrum of QDs stabilized with OA has shown the presence of characteristic vibration bands of alkyne and carboxylic acid dimers (1455 cm^{-1} - C-H stretching, 1709 cm^{-1} - C=O stretching, 2974 cm^{-1} - O-H stretching) and absent of TOPO characteristic vibration bands, i.e aliphatic ether (1162 cm^{-1} - C-O stretching). Analysis of QD optical spectra presented in Figure 1.b has demonstrated that QDs stabilized with OA molecules only are characterized by a bit higher energy excitonic band than as-synthesized QDs.
QDs stabilized with OA are characterized by 1.3% Photoluminescent Quantum Yield (PL QY) in toluene because of a lot of trap states on their surface. Therefore, these QDs are an excellent model for studying the correlation between efficiency of photoinduced processes on the QD surface and energy/charge transfer in Graphene/QDs hybrid structures.

S2. Characterization of the Graphene/QDs hybrid structures

S2.1 Scanning Electron Microscopy

Figure S2 shows SEM images of QDs and MLG layer onto a glass slide formed by L-B method (see Section 1.3 for details). Analysis of the SEM images has shown QDs formed good quality monolayer's film on the slide with a few inclusions with two monolayers of QDs.

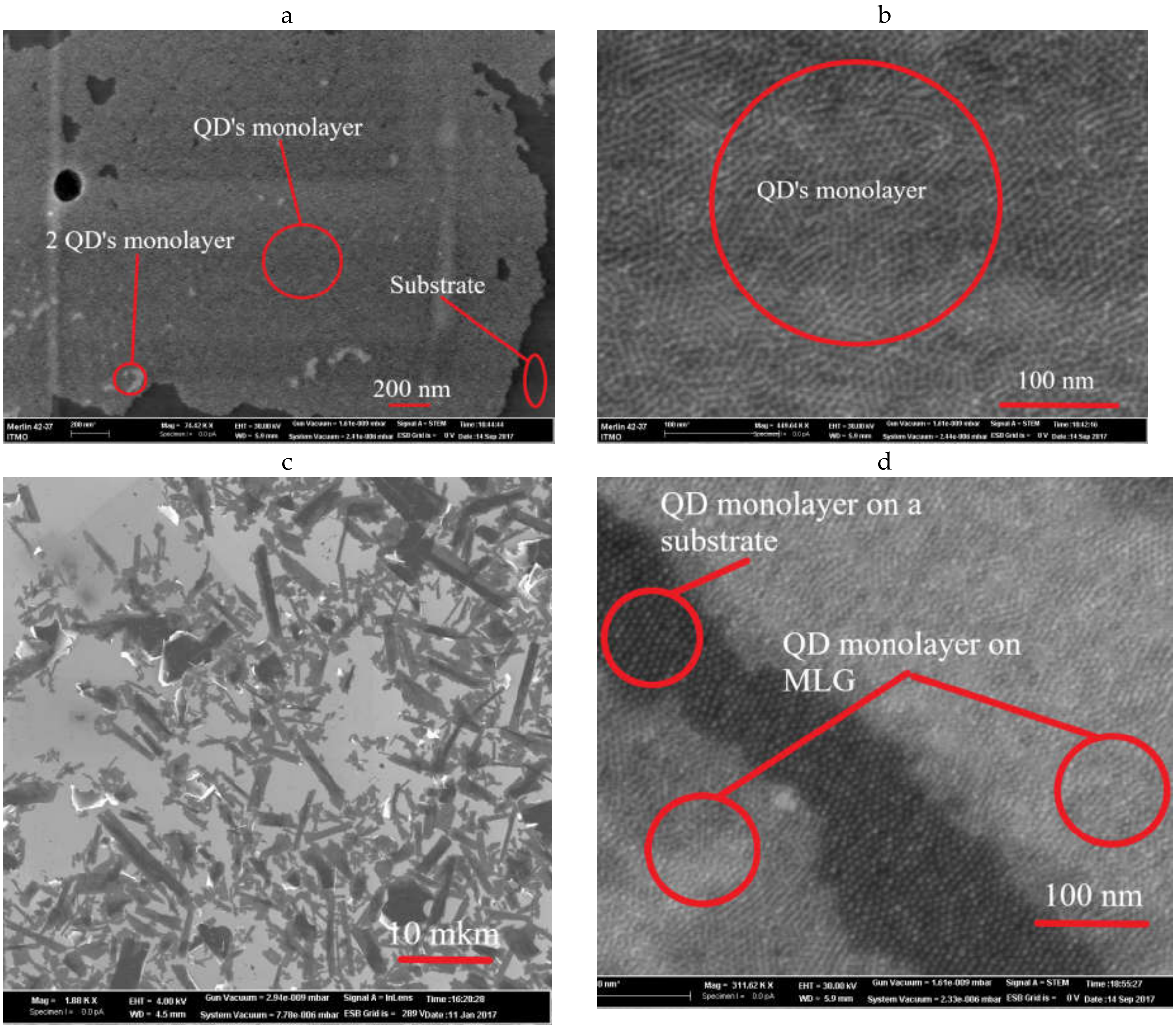

Figure S2. SEM images of a QD layer (a,b), MLG layer structure (c) and MLG/QD hybrid structure (d) with 74000x (a), 450000x (b), 1880x (c) and 310000x magnification (d) formed on a glass slide using the Langmuir-Blodgett method

S2.2 Atomic Force Microscopy

Figure S3 presents AFM image and the height profile of the QD film. It is clearly shown that the film mostly consists of one monolayer and small inclusions of areas with two or three QD monolayers formed during the transfer of the film from the water surface to the substrate. Therefore, it can be concluded that the Langmuir-Blodgett method allows the formation of QD monolayers.

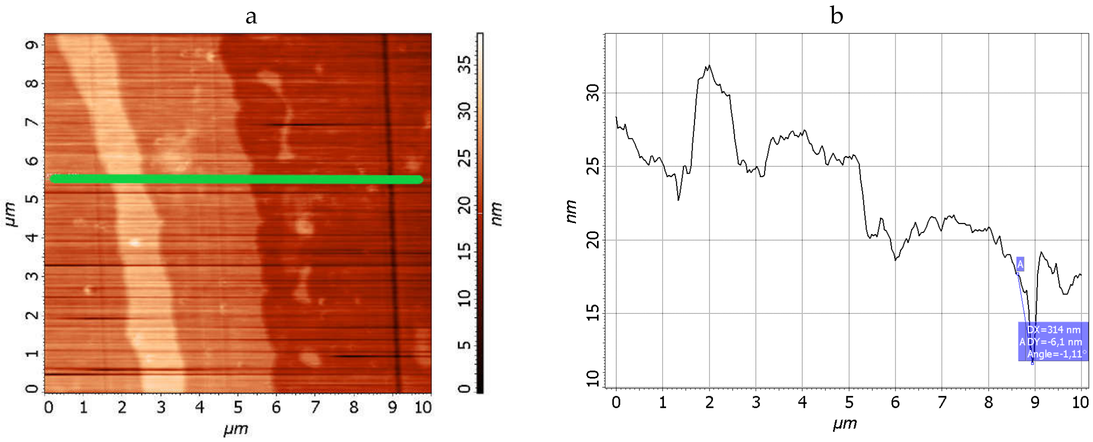

Figure S3. AFM image of 5.5 nm QD monolayer formed using the Langmuir-Blodgett technique (a) and the height profile of the QD monolayer(b)

S2.3. Confocal Luminescent Scanning Microscopy

Figure S4 presents PL images of QD monolayer (a) and Graphene/QD hybrid structure (b) and PL spectra (c) samples. The analysis of luminescent images has shown that the QD morphology is an island type on an area of about 800 μm^2. Small defects and film breaks are also caused by the transfer of a QD monolayer from the surface of the water to a dielectric substrate.

a

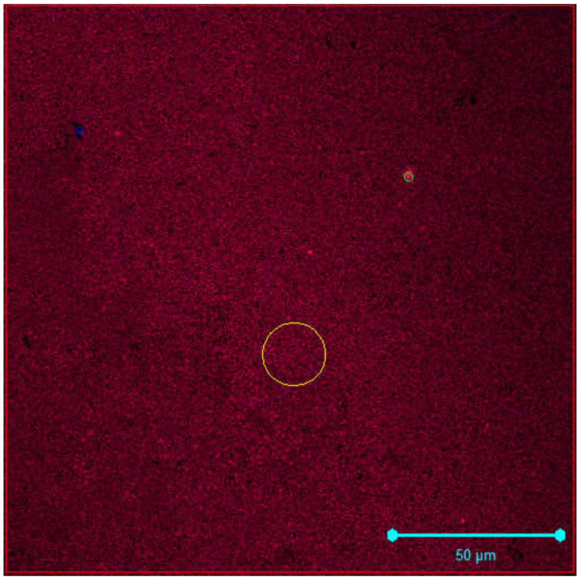

b

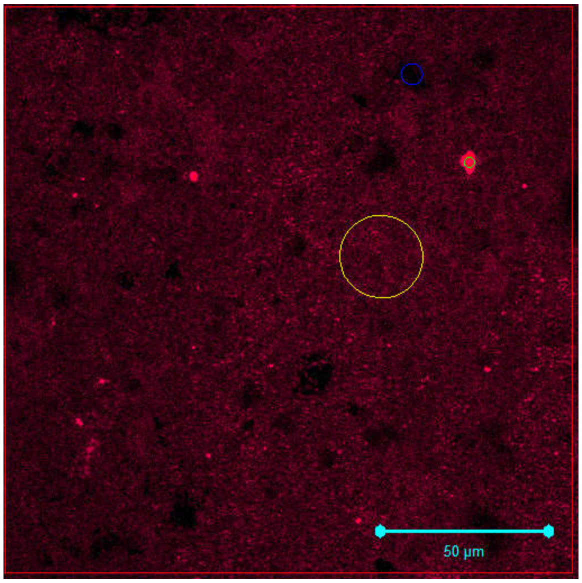

c

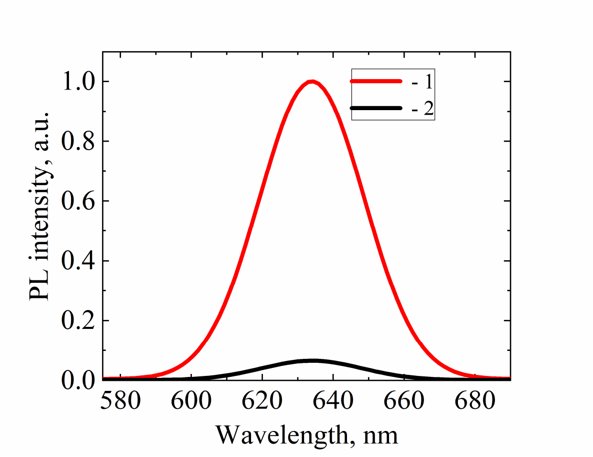

Figure S4. PL images of 5.5 nm CdSe QD monolayer on the dielectric slide (a) and CdSe QDs monolayer on graphene (b), and QD PL spectra from the respective areas (c), where curve 1 and 2 are QD PL spectra on dielectric slide and graphene layers, respectively. 405 nm laser excitation, 20x0.4 objective were used.

Analysis of PL images of QD monolayer and Graphene/QD structures presented in Figure 4 has shown that PL intensity of QDs has dramatically decreased in Graphene/QD structures due to efficient charge/energy transfer from QDs to Graphene [2-4]

S3.1. Calculation of photoactivation processes efficiency

Analysis of the photophysical properties of QDs on a dielectric substrate and on graphene showed that when a total radiation dose of 72 J was reached, the average decay time of QD luminescence on a dielectric substrate increased by ~ 35%, whereas in quantum dots that are part of hybrid structures, the increase in the decay time QD luminescence was ~ 17% (According to a Figure S5C,D). Such behaviour during photoactivation of quantum dots is caused by the presence of an effective channel of energy / charge carriers transfer from the quantum dot to graphene nanobelts. This, in turn, leads to the fact that quantum dots on a dielectric substrate are more actively exposed to the photoactivation effect than quantum dots that are part of hybrid structures with graphene nanobelts when exposed to a laser with a wavelength of 405 nm.

A more detailed component-wise analysis of the effect of photoactivation on the photophysical properties of QDs on a dielectric substrate and on graphene is presented in Figure S5.A and S5.B, respectively.

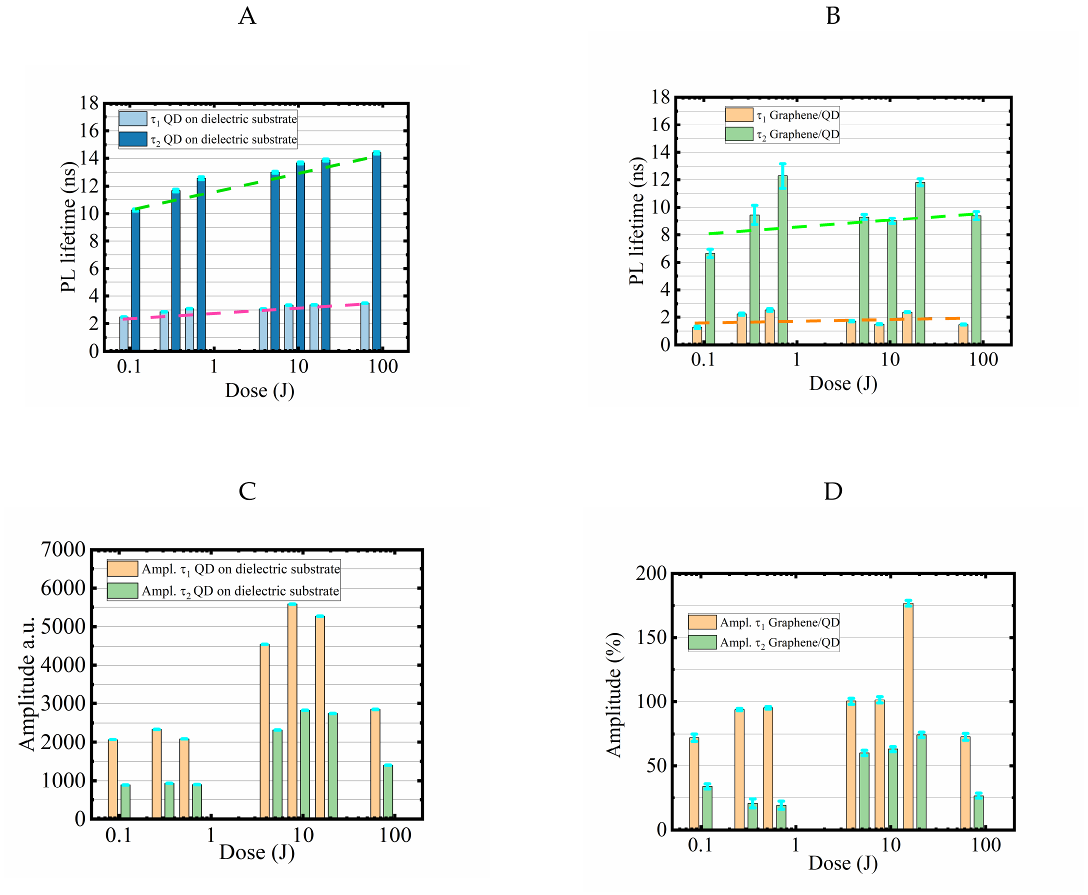

Figure S5. Analysis of the decay times (Figure S5 A,B) and amplitude (Figure S5 C,D) of the luminescence of QDs by component on a dielectric substrate (Figure S5.A,C) and as part of hybrid structures with graphene (Figure S5.B,D)

The analysis of the luminescence decay time of QDs on a dielectric substrate and the graphene / QD hybrid structures by components confirmed the increase in the luminescence decay time of the τ_1 and τ_2 QD fraction. In this case, for a quantitative analysis of the effectiveness of photoactivation, it is necessary to take into account the QD concentration of each fraction, and its dynamics over time. The calculation of the photoactivation efficiency of each QD fraction and the whole QD ensemble was carried out using Eqs. S1 and S2, respectively. The results of calculations of the photoactivation effectiveness before and after irradiation with UV radiation with a total dose of 72 J are presented in Table S1:

$$Q_i^{PA} = \left| \frac{A_i \cdot \dfrac{\tau_i}{\tau_{ri}}}{A_{0i} \cdot \dfrac{\tau_{0i}}{\tau_r}} - 1 \right| \cdot 100 \qquad \text{(S1)}$$

$$Q^{PA} = \left| \frac{\sum_{i=1}^{3} A_i \dfrac{\tau_i}{\tau_r}}{\sum_{i=1}^{3} A_{0i} \dfrac{\tau_{0i}}{\tau_r}} - 1 \right| \cdot 100 \qquad \text{(S2)}$$

where A_i - the concentration of excited QDs in this fraction is equivalent to amplitude *i*-th QD fraction (Figure S5C,D); τ_i - characteristic decay time of QD; $\tau_r = \frac{1}{k_r}$ - radiative relaxation time of the excited state 25 ns; the index "0" refers to the ensemble of QD before interacting with external irradiation.

Table S1. The effectiveness of the process of photoactivation of QD before and after exposure to UV radiation with a total dose of 72 J

	QD on dielectric substrate		Graphene / QD	
QD Fraction	τ_1 fraction of QD	τ_2 fraction of QD	τ_1 fraction of QD	τ_2 fraction of QD
Amplitude of the contribution of the QD fraction to luminescence before photoirradiation [1], counts	2074	893	72	34
The amplitude of the contribution of the QD fraction to the luminescence after photoirradiation [1], counts	2848	1404	73	27
The decay time of the QD luminescence before photoirradiation [2], ns	2.5±0.1	10±0.3	1.3±0.1	9.4±0.3
The decay time of the QD luminescence after photoirradiation [2], ns	3.5±0.2	14±0.5	1.5±0.1	6.6±0.3
Efficiency of photoactivation of the QD fraction Q^{PA_i} [3], %	92	115	17	11
Efficiency of photoactivation of the whole QD ensemble [4], %	106		12.5	

[1]- The amplitude of the contribution of each QD fraction on the dielectric substrate and in the composition of the hybrid structures with graphene are presented in Figure S5 C,D

[2]- The decay time of the luminescence of each fraction of QDs on a dielectric substrate and in the composition of hybrid structures with graphene is presented in Figure S5 A,B

[3]- The effectiveness of the process of photo-activation of QD for each fraction is calculated using Eq. S1

[4]- The effectiveness of the process of photoactivation of the entire QD ensemble is calculated using Eq. S2

As can be seen from Table S1, in the case of QDs on a dielectric substrate, the photoactivation efficiency is around ~ 106%, while for quantum dots on graphene, the photoactivation efficiency is reduced by an order of magnitude. Effectiveness more than 100% can be explained by the presence of completely extinguished (dark) QDs in the colloidal solution and monolayer. Presence of such dark QDs couldn't be registered through luminescence measurements, but such QDs still can be switched to luminescence state. Which causing the effect when total number of observed QDs are not the same before and after photoactivation. These data are in good agreement with the data on the QD luminescence quenching efficiency caused by the interaction between QDs and Graphene nanobelts (presented in Table 2). The

efficiency of this channel is ~ 98%, while the decrease in the efficiency of QD photoactivation, when switching to hybrid structures with Graphene, is ~ 94%. The results obtained for the entire ensemble of QDs considered in this work confirm that the presence of an effective channel of energy / charge carriers transfer from QDs to an external acceptor leads to a decrease in the efficiency of photoactivation.

References

[1] - Reiss P, Bleuse J, Pron A. Highly luminescent CdSe/ZnSe core/shell nanocrystals of low size dispersion. Nano letters. 2002 Jul 10;2(7):781-4.

[2] - Gromova YA, Reznik IA, Vovk IA, Rackauskas S, Alaferdov AV, Orlova AA, Moshkalev SA, Baranov AV, Fedorov AV. Photoinduced conductivity enhancement in quantum dot/multilayer graphene nanostructures. MRS Online Proceedings Library Archive. 2015;1787:15-9.

[3] - Reznik IA, Gromova YA, Zlatov AS, Baranov MA, Orlova AO, Moshkalev SA, Maslov VG, Baranov AV, Fedorov AV. Hybrid structures based on quantum dots and graphene nanobelts. Optics and Spectroscopy. 2017 Jan 1;122(1):114-9.

[4] - Gromova Y, Alaferdov A, Rackauskas S, Ermakov V, Orlova A, Maslov V, Moshkalev S, Baranov A, Fedorov A. Photoinduced electrical response in quantum dots/graphene hybrid structure. Journal of Applied Physics. 2015 Sep 14;118(10):104305.
